# Whole-exome sequencing identified a homozygous BRDT mutation in a patient with acephalic spermatozoa

**DOI:** 10.18632/oncotarget.15251

**Published:** 2017-02-10

**Authors:** Lin Li, Yanwei Sha, Xi Wang, Ping Li, Jing Wang, Kehkooi Kee, Binbin Wang

**Affiliations:** ^1^ Center for Stem Cell Biology and Regenerative Medicine, Department of Basic Medical Sciences, School of Medicine, Tsinghua University, Beijing, 100084, China; ^2^ Reproductive Medicine Center, Xiamen Maternal and Child Health Care Hospital, Xiamen, 361005, Fujian Province, China; ^3^ Center for Genetics, National Research Institute for Family Planning, Haidian, Beijing, 100081, China; ^4^ Department of Medical Genetics and Developmental Biology, School of Basic Medical Sciences, Capital Medical University, Beijing, 100069, China

**Keywords:** acephalic spermatozoa, whole-exome sequencing, BRDT, mutation, RNA-sequencing

## Abstract

Acephalic spermatozoa is a very rare disorder of male infertility. Here, in a patient from from a consanguineous family, we have identified, by whole-exome sequencing, a homozygous mutation (c.G2783A, p.G928D) in the *BRDT* gene. The gene product, BRDT, is a testis-specific protein that is considered an important drug target for male contraception. The G928D mutation is in the P-TEFb binding domain, which mediates the interaction with transcription elongation factor and might affect the transcriptional activities of downstream genes. By RNA-sequencing analysis of cells expressing the *BRDT* mutation, we found the p.G928D mutation protein causes mis-regulation of 899 genes compared with *BRDT* wild-type cells. Furthermore, by Gene Ontology analysis, the upregulated genes in p.G928D cells were enriched in the processes of intracellular transport, RNA splicing, cell cycle and DNA metabolic process, revealing the underlying mechanism of the pathology that leads to acephalic spermatozoa.

## INTRODUCTION

Acephalic spermatozoa (Human Phenotype Ontology, HP:0012869), also known as decapitated or pinhead sperm, is one extremely rare type of oligoasthenoteratozoospermia (OAT) in male infertility, and is defined as spermatozoa without heads in the ejaculates [1]. There have been very few reports of this disorder [2–5]. The uniform pattern of acephalic spermatozoa suggests that the phenotype has a genetic origin [6]. However, the molecular mechanism of this disorder was unclear and, recently, one study firstly reported that biallelic *SUN5* mutations caused acephalic spermatozoa in 47.06% of affected individuals [7].

The BRDT protein contains two bromo-domains, which are conserved domains involved in the recognition of H4 acetylated residues in histones [8–10]. The *BRDT* gene is testis-specific: it is expressed in spermatocytes, round spermatids, elongated sperm and mature sperm in humans [11, 12]. In the mouse, *Brdt* deficiency of the first bromo domain caused infertility, with low sperm number and reduced sperm motility, malformed heads and tails [13, 14]. Transcriptional analysis of *Brdt* knock-out mice revealed that Brdt could activate expression of 1872 testis-specific genes, and at the same time inhibit expression of 1155 genes [15]. Thus the function of BRDT is correlated with transcription and chromatin remodeling [16–18]. Based on the properties of this protein, BRDT was considered as an important drug target for male contraception [19]. One study found that single-nucleotide polymorphism (SNP) rs3088232 in *BRDT* was associated with male infertility among Albanians and Macedonians [20]. However, another study that consisted of 276 azoospermic and 182 fertile men of Arab and Jewish descent, demonstrated no association between *BRDT* rs3088232 and infertility [21]. Another Chinese group analyzed 361 men with non-obstructive azoospermia (NOA) and 368 fertile controls, and they could not find any *BRDT* variants associated with NOA susceptibility [22]. Thus, the association of *BRDT* with male infertility is inconclusive.

Here, we report a patient with acephalic spermatozoa in a consanguineous family. By whole-exome sequencing (WES), we found the patient inherited a homozygous missense mutation in *BRDT*. Using transcriptional analysis, we demonstrated that this mutation resulted in mis-regulation of expression of 899 genes compared with wild-type BRDT. Gene Ontology (GO) analysis of the 899 genes suggested a potential underlying mechanism of the disease pathology. This is the second study to report a genetic alteration that causes acephalic spermatozoa.

## RESULTS

### Whole-exome sequencing analysis of a patient with acephalic spermatozoa

**Figure 1:**
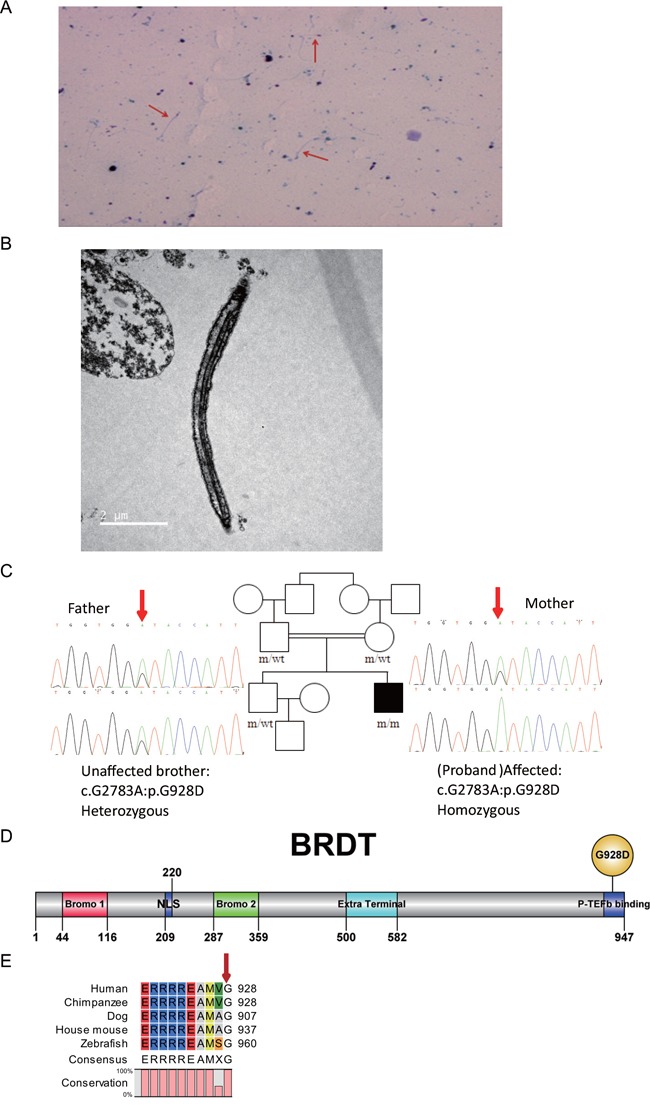
BRDT mutation in a patient with acephalic spermatozoa **A**. Papanicolaou staining. Red arrows indicate acephalic sperm. **B**. Electron microscopy shows the structure of acephalic sperm. No mitochondria were observed in the mid-piece of the sperm tail. **C**. Patient with acephalic spermatozoa in a consanguineous pedigree. The affected family member (black square) carries a homozygous *BRDT* mutation. The patient's father, mother and elder brother all carry heterozygous *BRDT* mutations. The red arrows point to the mutation site. **D**. Domains and mutation site in the BRDT protein. The full-length protein is 947 amino acids (aa). Bromo 1 domain, aa 44-116 (red box); bromo 2 domain, aa 287-359 (green box); extra terminal domain, aa 500-582 (blue box). The G928D mutation is located in the C terminal of the BRDT protein. **E**. Alignment of BRDT proteins from different species. The G928 site of human BRDT was 100 % conserved in the aligned sequences.

A patient with acephalic spermatozoa (Figure 1A and 1B) was recruited to this study. Electron microscopy showed that the acephalic spermatozoa did not contain any mitochondria (Figure 1B). Pedigree analysis suggested an autosomal recessive mode of inheritance associated with acephalic spermatozoa (Figure 1C). Thus, we focused on homozygous mutations identified in the patient by whole-exome sequencing. After filtering out polymorphisms with allele frequency greater than 1% in the dbSNP, 1000Genomes, ESP6500 and/or our inhouse database, we compiles a list of genes harboring homozygous mutations (Supplementary Table 1). Among these genes, only *BRDT* exhibited testis-specific expression, therefore, we hypothesized that the homozygous mutation in *BRDT* gene was associated with acephalic spermatozoa. By means of Sanger sequencing, the homozygous mutation in *BRDT* (NM_207189:exon19:c.G2783A:p.G928D), was confirmed in the patient (Figure 1C). This information has been deposited in the online human variation database LOVD3: http://databases.lovd.nl/shared/variants/0000116019#00024141. The patient's father, mother and elder unaffected brother all carried a heterozygous mutation in *BRDT* (Figure 1C).

### *In silico* analysis of the p.G928D mutation

*In silico* analysis predicted that the *BRDT* p.G928D mutation (abbreviated as G928D) is most probably a disease-associated mutation (Table 1). The allele frequency of c.G2783A in the East Asian population was only 0.0001 in the ExAC database (http://exac.broadinstitute.org/), which is consistent with the extreme rarity of acephalic spermatozoa. G928D is located in the P-TEFb binding domain in the C-terminal of the BRDT protein (Figure 1D). The P-TEFb binding domain mediates the interaction with transcription elongation factor and might affect the transcriptional activities of downstream genes [23, 24]. The glycine located at amino acid 928 in human BRDT is 100% conserved between different species from human to zebrafish (Figure 1E), indicating the functional importance of the G928 site. Thus we hypothesized that the G928D mutation might affect the transcriptional activities of BRDT. To test this hypothesis, we introduced the G928D-encoding mutation into *BRDT* and expressed wild-type (WT) BRDT and the G928D mutant in 293FT cells, respectively. Western blot analysis demonstrated that the expression level of the G928D mutant was similar to the WT BRDT protein (Supplementary Figure 1A), which suggested that the G928D mutation did not affect the steady state of BRDT protein.

**Table 1:** *In silico* analysis of *BRDT* mutation

mutation	Amino acid change	Polyphen-2^a^	SIFT^b^	Mutation Taster^c^	SNPs&GO^d^
c.G2783A	p.G928D	Probably-damaging (0.987)	Damaging (0)	Disease causing (0.99)	Disease (1)

^a^Polyphen-2 (http://genetics.bwh.harvard.edu/pph2/). Prediction Scores range from 0 to 1 with high scores indicating probably or possibly damaging.

^b^SIFT, i.e., Sorting Intolerant From Tolerant (http://sift.jcvi.org/). Scores vary between 0 and 1. Variants with scores close or equal to 0 are predicted to be damaging.

^c^Mutation Taster (http://www.mutationtaster.org/). The probability value is the probability of the prediction, i.e., a value close to 1 indicates a high ‘security’ of the prediction.

^d^SNPs&GO (http://snps.biofold.org/snps-and-go/). Reliability Index (RI) has a range from 0 to 10.

### RNA-sequencing analysis of p.G928D mutation cells

**Figure 2:**
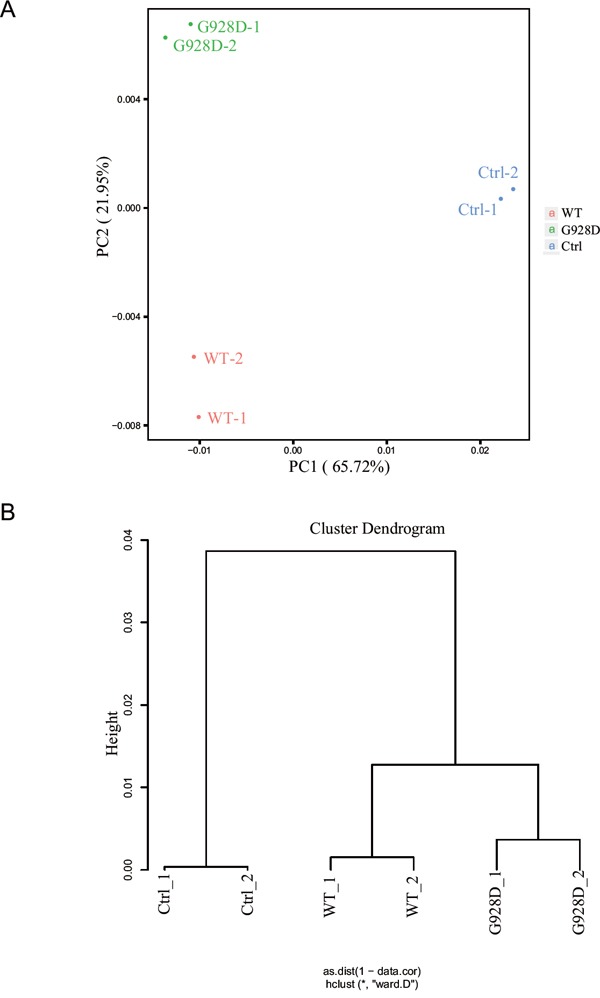
The genome-wide expression profile of G928D-BRDT cells differd from WT-BRDT cells **A**. Principal Component Analysis (PCA) of the RNA-sequencing result. NTERA-2 cells transduced with WT BRDT, G928D BRDT or GFP (Ctrl) lentivirus were selected with blasticidin for 3 days. After selection, these cells were collected (in duplicate) for RNA-sequencing analysis. **B**. Hierarchical clustering analysis of RNA-sequencing data.

**Figure 3:**
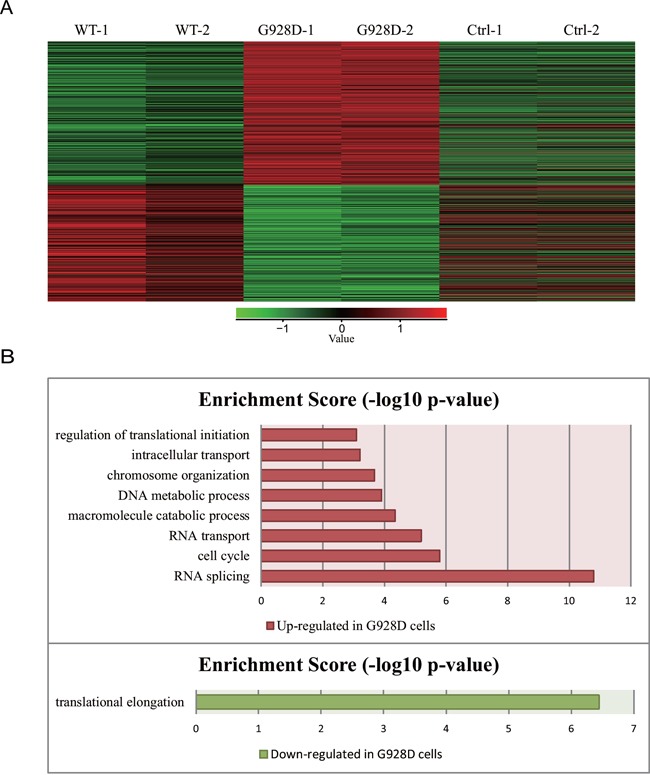
899 genes were differentially expressed between G928D and WT cells **A**. Heatmap data from the RNA-sequencing analysis. 899 genes were significantly differentially expressed between G928D and WT cells (experiment conducted in duplicate, FDR<0.01). Red and green indicates expression at relatively high and low levels, respectively. **B**. Gene Ontology (GO) analysis of the 899 genes, which described the biological processes of the up-regulated or down-regulated genes in G928D cells compared with WT cells. *p*-value is the adjusted *p*-value (FDR). WT=wild-type BRDT.

We next wanted to examine whether gene expression was affected by the G928D mutation. NTERA-2, a male teratocarcinoma cell line, was transduced with lentiviral overexpression vectors carrying GFP (negative control), WT BRDT or G928D BRDT. The BRDT mRNA expression level was similar between G928D and WT cells (Supplementary Figure 1B). We examined the expression of *NPC1, OAZ3, PPP1CC, SPATA6* and *STK36* by quantitative real time PCR (q-PCR). These genes’ were chosen as mice deficient for these genes exhibited acephalic spermatozoa. However, there was no significant difference in the expression level of any these genes between WT and G928D cells (Supplementary Figure 1C). Thus, transcriptome analysis was employed to examine the genome-wide expression profiles in G928D and WT cells. By principal component analysis (PCA) of RNA-sequencing data, we found that WT and G928D were similar to each other, but different from control cells in PC1 (Figure 2A). However, G928D cells were markedly different from WT BRDT cells in PC2 (Figure 2A). Hierarchical clustering analysis also demonstrated that the expression of genes’ in G928D cells was different than that in WT BRDT cells (Figure 2B). Furthermore, 899 genes were found to be significantly differently expressed (FDR<0.01) between WT BRDT and G928D cells (Figure 3A). By GO analysis, we found some biological processes, for example RNA splicing, cell cycle, RNA transport, DNA metabolic process and intracellular transport, were enriched in the genes up-regulated in G928D cells (Figure 3B). Only one biological process, translational elongation, was enriched in the genes down-regulated in G928D cells (Figure 3B). This alteration in expression of a wide range of genes might be associated with the pathology of acephalic spermatozoa.

## DISCUSSION

We report here a homozygous mutation, p.G928D, of the *BRDT* gene in a patient with acephalic spermatozoa (Figure 1C). By functional analysis of the mutation, we found 899 genes were differentially expressed between G928D and WT BRDT cells (Figure 3A). GO analysis also showed that genes related with RNA splicing, cell cycle, RNA transport, DNA metabolic process and intracellular transport were up-regulated in G928D cells (Figure 3B). This GO result was consistent with a previously reported gene expression profile observed in the sperm of asthenozoospermic patients [25]. Therefore, the differentially expressed genes induced by G928D BRDT might explain the phenotype of low sperm motility in our acephalic spermatozoa patient.

In 1984, Bacetti *et al*., found the malformation of acephalic spermatozoa was due to an overproduction of vesicles by the Golgi complex in the region between the centrioles and nucleus [3]. In our study, we found intracellular transport related genes were up-regulated in G928D cells, especially some genes involved in the endosome to trans-Golgi network process, such as *VPS54, GOPC, EEA1, KIF5B* and *ARL1*. So we proposed that up-regulation of these genes might be associated with the overproduction of Golgi complex, which might affect the attachment of the proximal centrioles to the nucleus [1], and might thereby influence the formation of head-neck attachment [1]. To our surprise, the knock-out mice of genes *VPS54* or *GOPC* presented with globozoospermia [26, 27], another type of sperm head abnormality, indicating a relationship in pathology between the acephalic spermatozoa and globozoospermia [6].

In our functional study, the G928D mutation was found to change the gene expression profile induced by BRDT. G928D is located in the P-TEFb binding domain in the C-terminal of the BRDT protein (Figure 1D) and the G928 site was highly conserved (Figure 1E). Previous studies have found that the P-TEFb binding domain mediates the interaction with transcription elongation factor and might affect the transcriptional activities of downstream genes [23, 24]. Additionally, expression of a C-terminal tagged Brdt in mice resulted in a severe infertility phenotype [15]. Therefore, the G928D mutation might affect the ability of the C-terminal of the BRDT protein to recruit P-TEFb complex to the acetylated chromatin [15, 23], and change the transcriptional activities of the BRDT protein. Further studies need to validate whether the interaction between BRDT and the P-TEFb complex was enhanced or attenuated by the G928D mutation.

Although a recessive mode of inheritance is commonly associated with loss-of-function, our genome wide gene expression analysis suggested that the G928D mutation resulted in a gain-of-function; therefore we can categorize it as a recessive gain-of-function mutation. The recessive gain-of-function, though rare, has been reported previously in acute myeloid leukemia [28] and in the genetics of Arabidopsis [29] (http://openscholarship.wustl.edu/vol9_iss1/114). In the transcriptional analysis of the G928D mutation, the level of gene expression up-regulation by the mutant was not very high (1.5 fold change), so we propose that the effect of one mutant allele does not reach a threshold level required to bring about the disease phenotype, whereas two mutant alleles can reach the threshold level and result in acephalic spermatozoa. The pathogenic effect related with threshold level in the recessive gain-of-function is similar to the threshold level in haploinsufficiency [30], the only difference is that the former is gain-of-function while the latter is loss-of-function. The genetic mode of recessive gain-of-function has also been proposed by Bob Horvitz in the analysis of lin-1 “Vul/Let” alleles in worm genetics (http://www.wormbook.org/wli/wbg13.3p101/).

Taken together, our study demonstrated for the first time a homozygous mutation p.G928D of the *BRDT* gene, which exerted a significant effect on the gene expression profile and might lead to acephalic spermatozoa by affecting the processes of RNA splicing, cell cycle, DNA metabolic process and intracellular transport. This finding provides researchers and clinicians with a better understanding of the pathology and molecular mechanism of acephalic spermatozoa.

## MATERIALS AND METHODS

### Patients

The acephalic spermatozoa patient was recruited from Xiamen Maternal and Child Health Care Hospital. Acephalic spermatozoa syndrome is diagnosed if the patient's ejaculate contains mostly sperm flagella without heads. This patient was infertile and had 99.5% acephalic spermatozoa, with 7.6×10^6^/mL sperm density and 0.5% sperm motility. The acephalic spermatozoa did not contain any mitochondria by electron microscopy. The chromosomal karyotype of the patient was normal 46; XY, and no deletion was found in the Y chromosome. The hormones levels of the patient were normal as follows: follicle stimulating hormone (FSH), 3.65 mIU/mL; luteinizing hormone (LH), 2.16 mIU/mL; testosterone (T), 5.01 ng/mL; estradiol (E2), 46 pg/mL; prolactin (PRL), 4.98 ng/mL. The patient's brother was fertile. This study was approved by the Ethics Committee of National Research Institute for Family Planning. Written informed consent was obtained and then 5 ml of peripheral blood was collected from each participant.

### Exome sequencing

Exome sequencing was carried out as previously described [31]. Variants fulfilling the following criteria were retained for subsequent analyses: (i) missense, nonsense, frame-shift or splice site variants; (ii) absent or rare (frequency < 1%) in dbSNP (http://www.ncbi.nlm.nih.gov/snp/), 1000 Genomes (http://browser.1000genomes.org/index.html), Exome Aggregation Consortium (ExAC, http://exac.broadinstitute.org/) and in-house database.

### Vectors construction

The full-length human *BRDT* coding sequence (2844 base pairs, NCBI transcript ID NM_207189) was amplified by polymerase chain reaction (PCR) from a commercial plasmid (ORIGENE, RC222276) using specific primers. *BRDT* wild-type (WT) and c.G2783A (p.G928D) alleles were subcloned into pENTR-D-TOPO plasmid. Overlapping PCR was used to generate the p.G928D mutation described in this paper. Then the *BRDT* wild-type and p.G928D mutation sequence were recombined into p2k7 [32] vector using Gateway^®^ LR Clonase^®^ II (Thermo Fisher Scientific, USA) to create a lentivirus vector as we previously described [33]. The primers used for these methods are listed in Supplementary Table 2.

### Cell culture and plasmid transfection

293FT cells and NTERA-2 cells were cultured and passaged as previously described [34, 35]. 293FT cells were seeded on 6-well plates at a 30% cell confluency and transfected with 500 ng/well p2k7-*BRDT* wild-type, p.G928D variant or GFP plasmids using Lipofectamine 2000 (Thermo Fisher Scientific, USA), according to the manufacturer's protocol. p2k7-GFP plasmid is the negative control plasmid used in this study. Cells were cultured for approximately two days after transfection and then used in further experiments described below.

### Western blot analysis and real time quantitative PCR

Western blot analysis and real time quantitative PCR were performed as previously described [33]. The specific antibodies used in Western blot analysis are listed in Supplementary Table 3. Real time quantitative PCR primers for all gene sequences are listed in Supplementary Table 2. The statistical significance of the data was calculated using One-way ANOVA and Prism 5.0 software.

### Lentivirus production and transduction of NTERA-2 cells

Lentivirus was produced in 293FT cells by transient transfection of the p2k7-bsd-BRDT, p2k7-bsd-BRDT-G928D or p2k7-bsd-GFP plasmid with Vsvg and delta-8.9 packaging plasmids as previously described [33]. For the infection of NTERA-2 cells, the filtered lentivirus-containing supernatant was added onto the NTERA-2 cells, which were at 30 % confluence. After 6 hrs, NTERA-2 cell medium was added. One day after infection, blasticidin (Bsd, Thermo Fisher Scientific, USA) was added to the culture medium of NTERA-2 cells. Bsd selection was maintained for 3 days on NTERA-2 cells. Afterwards, RNA was extracted from these cells and analyzed by RNA-sequencing.

### RNA-sequencing and data analysis

RNA-sequencing (RNA-Seq) was performed by Annoroad Gene Technology Co., Ltd, Beijing, China. Briefly, after RNA quality examination, library preparation and construction was performed using NEBNext^®^ Ultra™ RNA Library Prep Kit for Illumina^®^ (#E7530L, NEB, USA), followed by library examination, library clustering and sequencing on the Illumina Hiseq 4000 platform. Read pairs with more than 10 % low-quality bases, adapter contaminant or artificial sequences introduced during the experimental processes were trimmed, and the cleaned reads were aligned to the human hg19 reference using Tophat (v2.0.12) with default settings [36]. Unique mapped reads mapped on each gene were counted using Htseq [37], and this was further used to quantify transcription levels (RPKM, reads per kilobase of transcript per million mapped reads) using the formula: RPKM=read × 10^9^ / (gene length × total reads). To assess the cell populations of all the RNA-Seq samples, the Pearson coefficients were computed using all genes expressed in more than two samples, and hierarchical clustering was performed using the hclust function, and “ward.D2” method in R. Principal Component Analysis (PCA) was performed using the same criterion with the prcomp function in R. Differential gene expression analysis was performed by using R Bioconductor, DESeq2 package, as previously described [38]. This procedure was based on a negative binomial distribution. Raw read counts were employed to build this model, and the false-discovery rate (FDR) was used to correct for multiple testing errors. Only the genes with significant p-values and FDR less than 0.01 were considered to be differentially expressed. The results for differentially-expressed genes were further presented using the matplotlib package in python. Gene ontology analysis was carried out using DAVID functional annotation bioinformatics microarray analysis (https://david.ncifcrf.gov/). The RPKM value of all genes in these 6 samples has been deposited in an Excel file in Supplementary Table 4.

## SUPPLEMENTARY MATERIALS FIGURES AND TABLES




