# Supplementary material for: Whole-exome sequencing identified a homozygous BRDT mutation in a patient with acephalic spermatozoa
Source: Oncotarget. 2017 Feb 10;8(12):19914–22. doi: 10.18632/oncotarget.15251 (PMC5386733; doi:10.18632/oncotarget.15251)
Supplement: Supplementary file 1 [file oncotarget-08-19914-s001.pdf]

Whole-exome sequencing identified a homozygous *BRDT* mutation in a patient with acephalic spermatozoa

SUPPLEMENTARY FIGURE AND TABLES

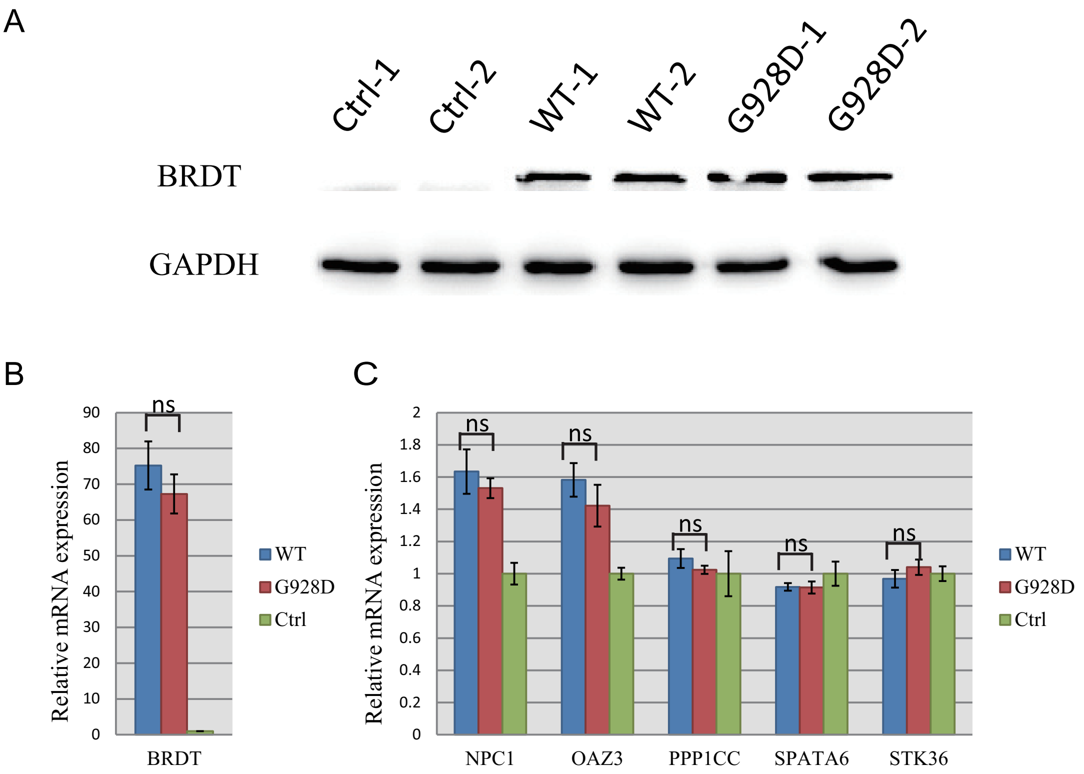

Supplementary Figure 1: A. Western blot analysis of BRDT protein expression. 293FT cells were transiently transfected with GFP (Ctrl), WT or G928D BRDT expression vector. GAPDH is the house keeping protein used in this western blot as a loading control. **B** and **C.** Quantitative real-time PCR (q-PCR) analysis of gene expression in WT, G928D and Ctrl cells. NTERA-2 cells were infected with WT, G928D BRDT or GFP (Ctrl) lentivirus and selected with blasticidin (to kill the uninfected cells) for 3 days. The mRNA expression is relative to *GAPDH*. Experiments were performed in triplicates. ns, not significant.

Supplementary Table 1: Homozygous sequence variants were found by whole-exome sequencing

Gene	Change	Annotation
ADAMTS7	nonsynonymous_SNV	NM_014272:exon21:c.G4496A:p.R1499Q,
ADAMTSL4	nonsynonymous_SNV	NM_019032:exon17:c.G2839A:p.V947M,
BCAP31	nonsynonymous_SNV	NM_005745:exon2:c.T53C:p.V18A,
BRDT	**nonsynonymous_SNV**	**NM_207189:exon19:c.G2783A:p.G928D,**
DMD	nonsynonymous_SNV	NM_004009:exon17:c.C2084G:p.A695G,
GABPB2	nonsynonymous_SNV	NM_144618:exon6:c.C701T:p.A234V,
GAPVD1	nonsynonymous_SNV	NM_015635:exon19:c.G3232A:p.A1078T,
LOC100507003	nonsynonymous_SNV	NM_001195256:exon1:c.C67T:p.P23S,
MYBBP1A	nonsynonymous_SNV	NM_014520:exon1:c.T104C:p.F35S,
PHKG1	nonsynonymous_SNV	NM_006213:exon8:c.C773T:p.S258L,
PIP5K1A	nonsynonymous_SNV	NM_003557:exon1:c.C68A:p.S23Y,
SLC12A7	nonsynonymous_SNV	NM_006598:exon19:c.C2498T:p.S833L,
TMEM26	nonsynonymous_SNV	NM_178505:exon1:c.G25T:p.A9S,
CHRNA3	nonsynonymous_SNV	NM_000743:exon5:c.C1112T:p.P371L,
GPR144	nonsynonymous_SNV	NM_001161808:exon7:c.G1301A:p.R434H,
HKDC1	nonsynonymous_SNV	NM_025130:exon14:c.A1984C:p.M662L,

Supplementary Table 2: DNA Primers used in this study

Gene	Forward Primer	Reversed Primer	Experiment
GAPDH	TGTTGCCATCAATGACCCCTT	CTCCACGACGTACTCAGCG	Q-PCR
BRDT	TCCCTTGAACGTGGTACAGG	GCAGGAGTTGTTGTATCTGCT	Q-PCR
NPC1	GTCCAGCGCAGGTGTTTTC	GCCGAACATCACAACAGAGAC	Q-PCR
OAZ3	TATCCCATATCAGGCCTTGG	ACCTCTGTCGTTCCGATCAT	Q-PCR
PPP1CC	GCGATGGCGGATTTAGATA	GCTGGACATTCTTACCAGGC	Q-PCR
SPATA6	CATCAGGCTCATCAAAGACATT	GGGTCACTGTCATAGGCAGA	Q-PCR
STK36	CGGAGCACTGATGTAGTGGA	AATAGGCACAGGCTCAGTGG	Q-PCR
BRDT PCR with NotI or AscI restriction enzyme sites	gactgcggccgcaccATGTCTCTGCC AAGTCGACAAACAG	gactggcgcgccTTAATCAAAGTT GTTTTCAAACATTGTCATA	Vectors Construction
BRDT-G928D overlap PCR	GAGAAGCAATGGaGGGTACCA TTGATATGA	TCATATCAATGGTAtCCA CCATTGCTTCTC	Vectors Construction

Supplementary Table 3: Antibodies used in Western Blot

Primary/ Secondary antibodies	Source	Western Dilution	Cat no.
BRDT	Rabbit polyclonal	1:1000	AP7115a, ABGENT
GAPDH	Mouse monoclonal	1:2000	60004-1-Ig, Proteintech Group
Goat Anti-Rabbit IgG (H+L)		1:1000	ZB-2301, ZSGB-BIO
Goat Anti-Mouse IgG (H+L)		1:1000	ZB-2305, ZSGB-BIO

Supplementary Table 4: Expression levels of RefSeq genes in G928D-BRDT or WT-BRDT over-expression cells

See Supplementary File 1
